# Supplementary material for: EEG Sonification improves sleep staging performance in novice stagers
Source: PLoS One. 2025 Sep 11;20(9):e0329711. doi: 10.1371/journal.pone.0329711 (PMC12425293; doi:10.1371/journal.pone.0329711)
Supplement: Attachment — Submitted filename: Re.docx [file pone.0329711.s001.docx]

**Re:** **EEG Sonification Improves Sleep Staging Performance in Novice Stagers**

**General comments**

Manuscript “EEG Sonification Improves Sleep Staging Performance in Novice Stagers” aimed at investigating whether adding an auditory representation (sonification) of the EEG to a standard visual representation could improve sleep staging performance. Additionally, the study examines if this method reduces workload. The authors found that adding sonification did not result in improvements in accuracy, speed, or workload for the entire subject group .However we found sonification improved accuracy for the least experienced participants. This study explores an interesting and promising concept. Below are some suggestions that should be considered by the authors of the manuscript:

**Specific comments**

**Abstract**

1. In the abstract, also mention the classification of groups based on the level of experience.

**Methods**

2. How many groups did you have in total? Please provide a detailed explanation of how the groups were divided based on the level of experience and the reception of the Sanifcation sound.

**Introduction**

1. The first paragraph of the “Manual Sleep Staging” section needs more references.

2. Hypothesis 1 should be written in full. In which aspect does it perform better?

**Statistical analysis**

1. Why is only the prior year experience of the participants considered? It seems that if the overall experience of the participants is taken into account, the accuracy of the study would be higher.

**Discussion**

Overall the discussion is not well written.

1. The consequences of the findings in relation to the hypotheses proposed in this study should be clarified with the existing literature.

2. How do you justify the effectiveness of Sanification for novices? You need to reference relevant articles on this.

3. Paragraph 5 is not relevant to this section. It is suggested that it be briefly mentioned in the Method section.
